# Effects of N361 Glycosylation on Epidermal Growth Factor Receptor Biological Function

**DOI:** 10.3390/cancers18030474

**Published:** 2026-01-31

**Authors:** Dennis Lam, Brandon Arroyo, Ariel N. Liberchuk, Jessica Das, Leonard J. Ash, Khizr M. Khan, Jayati Mondal, Andrew L. Wolfe

**Affiliations:** 1 Department of Biological Sciences, Hunter College, City of University of New York, New York, NY 10065, USA; 2 New York Research and Mentoring for Postbaccalaureates (NY-RaMP) Program, Hunter College, City of University of New York, New York, NY 10021, USA; 3 Maximizing Access to Research Careers Program, Hunter College, City University of New York, New York, NY 10065, USA; 4 Macaulay Honors College, Hunter College, City University of New York, New York, NY 10065, USA; 5 Molecular, Cellular, and Developmental Biology Ph.D. Subprogram, The Graduate Center, City University of New York, New York, NY 10016, USA; 6 Yalow Scholars Program, Hunter College, City of University of New York, New York, NY 10065, USA; 7 Damon Runyon Cancer Research Foundation, Scholars Program for Advancing Research and Knowledge, New York, NY 10006, USA; 8 Department of Pharmacology, Weill Cornell Medicine, New York, NY 10065, USA; 9 Biochemistry Ph.D. Program, The Graduate Center, City University of New York, New York, NY 10016, USA

**Keywords:** glycosylation, epidermal growth factor receptor (EGFR), cell biology, proliferation, epidermal growth factor (EGF), ligand, non-small cell lung cancer (NSCLC), breast cancer, co-localization

## Abstract

Background: Epidermal growth factor receptor (EGFR) is a transmembrane tyrosine kinase that is frequently post-translationally modified by glycosylation. In cancer, EGFR amplifications and hotspot mutations such as L858R that promote proliferation have been detected in a significant fraction of non-small cell lung carcinomas and breast adenocarcinomas. Analyses of disruptions to glycosylation pathways in cancer cells identified EGFR glycosylation at residue N361 as one of the most heavily impacted sites. Methods: We stably expressed a glycosylation-deficient mutant EGFR, N361A, with or without the oncogenic EGFR mutation L858R in cells. Proximity ligation assays were employed to study the effects of the glycosylation mutant on the co-localization of EGFR and HER2. The effects of the glycosylation-deficient mutant on the efficacy of agonists, such as EGF and amphiregulin, or antagonists, such as osimertinib and necitumumab, were defined using cell viability assays and immunoblots. Results: N361A increased the membrane localization and co-localization of EGFR with its binding partner HER2. The glycosylation-deficient mutation decreased cell proliferation, including proliferative responses to EGFR ligands. The mutant cells demonstrated reduced sensitivity to inhibition using the antibody inhibitor necitumumab, which inhibits EGFR by binding the extracellular domain. Conclusions: Disruption of glycosylation at N361, located near the ligand binding and dimerization regions, created a dominant negative form of EGFR, which non-productively co-localized with HER2, resulting in a blockage in proliferation. These findings underline the critical relevance of post-translational glycosylation modifications on EGFR function.

## Simple Summary

Activating genetic changes in the gene that encodes epidermal growth factor receptor (EGFR) frequently promote uncontrolled proliferation in lung cancer and breast cancer. Glycosylation modifications added to asparagines after protein translation can impact protein function. This study focuses on the impacts of a site on EGFR that is frequently N-glycosylated. We found that glycosylation-deficient mutation of this site promotes increased membrane localization and non-productive co-localization of EGFR with its binding partner HER2. Proliferation was reduced in mutant cells. The mutant EGFR was deficient at binding and responding to growth factor ligands that promote cell proliferation. Loss of this glycosylation site reduced the efficacy of the cancer therapeutic antibody necitumumab, which binds nearby. These findings underline the critical relevance of post-translational glycosylation modifications on EGFR function, cell biology, and responses to therapeutic agents.

## 1. Introduction

EGFR is a transmembrane tyrosine kinase that tranduces extracellular growth factor signals into cellular proliferation. EGFR is a member of the ErbB family of receptors, which includes its heterodimerization partner HER2. EGFR activity is regulated by the binding of agonist ligands, such as epidermal growth factor (EGF) or amphiregulin (AREG), to the extracellular domain, promoting dimerization that relies on an extracellular interface near the ligand binding site [1,2,3]. Ligand binding leads to increased dimerization, autophosphorylation of the carboxyl tail at Y1068 and other sites, activation of downstream cascades through the KRAS and mTOR pathways, and increased cell proliferation [4,5,6].

Activating point mutations and amplifications in EGFR are commonly detected in many types of malignancies, occurring in 15–35% of non-small cell lung cancer (NSCLC) and 4–14% of breast adenocarcinomas [7,8,9,10]. In NSCLC, the most common EGFR activating hotspot mutation is a leucine to arginine mutation at residue 858 (L858R), which occurs in 7% of cases [9,10]. L858 is located in the kinase domain, and its mutation to arginine promotes ligand-independent proliferation, making cells expressing EGFR L858R less dependent on EGF to induce downstream signaling [11]. EGFR L858R expression is sufficient to induce a cancerous phenotype in many systems.

Therapeutics inhibiting EGFR kinase activity, such as osimertinib (AZD-92921), are approved for clinical use against EGFR mutant NSCLC. Osimertinib is most effective against EGFR with the kinase domain hotspot mutations T790M and L858R [12]. Several therapeutic antibodies that target the extracellular domain of EGFR have been approved, including necitumumab to treat NSCLC. Necitumumab (IMC-11F8) binds to the extracellular domain of EGFR to inhibit dimerization, reduce affinity for ligands, and attract inhibitory immune cells [13,14,15].

Post-translational modifications (PTMs) on receptor tyrosine kinases can modify the ability of ligands to bind, dimers to interact, effectors to be activated, and cells to proliferate [16,17]. Glycosylation is a tightly regulated PTM where structured sugar groups are attached to target proteins, most commonly on asparagine for N-glycosylation and serine or threonine for O-glycosylation. Globally, sialylated and fucosylated N-glycans are more prevalent in cancer cells, particularly in metastatic cancer cell lines [18,19,20]. ErbB family members are heavily glycosylated, and several N-glycosylation sites have been detected on EGFR [21,22,23,24]. For instance, unglycosylated EGFR N603 mutants have increased propensity to dimerize without ligand, but are deficient at increasing cell survival in the absence of stimulatory ligand [25]. In addition to regulating ligand binding, glycosylation can also impact the ability of ErbB proteins to be bound by therapeutic antibodies [26]. Previous glycoproteomic and total proteomic data revealed that, in cancer cells with disrupted glycosylation, one of the most significantly changed sites was a family of N-glycosylations on EGFR at residue N361, sometimes referred to as N337 corresponding to the structure PDB ID: 7SYE, where the first 24 amino acids were truncated [27,28,29,30]. Knockdown of the glycosylation regulating enzyme UGP2 in KRAS-mutant cancer cells decreased EGF/EGFR-dependent activation of signaling cascades, including readouts of EGFR kinase activity that were dependent on EGFR kinase activity [27].

Glycosylation at EGFR N361 occurs in non-cancerous ovarian lines, lung adenocarcinoma, pancreatic ductal adenocarcinoma, and human epidermoid carcinoma cells [24,27,29,30,31,32,33]. In other ErbB family members, glycosylation at the homologous site to EGFR N361 is conserved in ErbB3 and ErbB4, but not in ErbB2 [24]. N361 sits at the edge of the receptor L domain near the junction with the furin-like cysteine-rich domain, forming one face of the extracellular EGF binding cavity [21,30]. Molecular dynamic simulations of glycosylation at N361 of EGFR showed large changes in side chains, differences that were magnified in the presence of EGF ligand [21,22,34,35]. These simulations also suggested that glycosylation sugars at N361 may interact with the opposing EGFR dimer [21,32]. However, the functional relevance of how the glycosylation of EGFR at N361 impacts EGFR protein and cellular behaviors remains unclear.

Here, we stably introduced an EGFR mutation incapable of being glycosylated at N361 into non-transformed MCF10A and HEK-293T (293T) cell lines. These lines were selected because they did not have pre-existing mutations constitutively activating the EGFR pathway. Using both in an otherwise wild-type context and in the context of the oncogenic EGFR L858R mutation, we determined that N361A increased co-localization with HER2, yet decreased proliferation, ligand response, and downstream signaling, suggesting that glycosylation deficiency at N361 promotes increased non-productive co-localization. We determined how a lack of this specific PTM impacted co-localization with binding partners, cell proliferation, response to ligands, downstream proliferative signaling, and efficacy of EGFR inhibitors that act on the extracellular or kinase domains.

## 2. Materials and Methods

### 2.1. Constructs and Stable Cell Lines

Initial plasmids from Addgene (Watertown, MA, USA) included EGFR wild-type (EGFR WT, RRID: Addgene_11011), pBABE puro EGFR L858R (RRID:Addgene_11012), and pBABE puro IRES-eGFP (RRID:Addgene_14430, described throughout the paper as “empty vector”) [36]. The introduction of N361A by site-directed mutagenesis on the pBABE puro was performed by Azenta Inc. (Watertown, MA, USA). All plasmid sequences were independently confirmed by Sanger sequencing.

Stable MCF10A and 293T cell lines overexpressing EGFR constructs or empty vector controls were created using viral packaging Phoenix-AMPHO cells (ATCC, Manassas, VA, USA, Cat# CRL-3213, RRID:CVCL_H716) transfected using a 4:1 ratio of plasmid of interest to psPAX2 (RRID:Addgene_12260) in Opti-MEM™ I, Reduced Serum Medium, GlutaMAX™ supplement (Gibco, Waltham, MA, USA, Cat# 51985034) using the Lipofectamine 3000 Kit (Thermo Fisher Scientific, Waltham, MA, USA, Cat# L3000015) following the manufacturer’s instructions. After at least 72 h of recovery, cell lines were twice selected with 4 µg/mL puromycin (Gibco Cat# A1113803), with each selection lasting 1 week until non-transduced parental control cells were eliminated.

MCF10A (RRID:CVCL_0598) cells were cultured and maintained in DMEM/F12 media (Gibco Cat# 11330057), 5% Horse Serum (Invitrogen, Waltham, MA, USA, Cat# 16050-122), and 1% Penicillin Streptomycin Glutamine (PSG) (Gibco Cat# 10378016), with the addition of 20 ng/mL EGF (Peprotech, Cransbury, NJ, USA, Cat# AF-100-15), 0.5 mg/mL hydrocortisone (Sigma-Aldrich, St. Louis, MO, USA, Cat# H-0888), 100 ng/mL cholera toxin (Sigma-Aldrich Cat# C-8052), and 10 µg/mL insulin (Sigma-Aldrich Cat# I-1882), as supplements to create their complete media. HEK-293T (293T, RRID:CVCL_0063) cells were maintained in DMEM (Gibco Cat# 11965092), 10% fetal bovine serum (FBS, R&D Systems, Minneapolis, MN, USA, Cat# S11550H), and 1% PSG as their complete media.

Cells were maintained at 5% CO_2_ in a 37 °C humidified incubator. All cells were counted using either a Countess 3 Automated Cell Counter (Invitrogen) or a hemocytometer. Stimulated media for both cell lines contained complete media with an additional 20 ng/mL EGF. All cell line identities were confirmed using short tandem repeat analysis (ATCC Cat# 135-XV). All cell lines tested negative for mycoplasma using MycoAlert® Mycoplasma Detection Kit (Lonza, Basel, Switzerland, Cat# LT07-318).

### 2.2. Viability Experiments

Live cells were plated at 5000 cells/well in sterile optical-bottom 96-well plates (Thermo Fisher Scientific Cat# 165306) in 200 µL of complete or stimulated media. For time-course experiments, relative viability was measured in triplicate by ATP quantification at 0 h, 24 h, 48 h and 72 h after treatment using CellTiter-Glo Assay kits (Promega, Madison, WI, USA, Cat# G7572) read on a Varioskan Lux (Thermo Fisher Scientific Cat# VLBL00GD2).

For dose courses, the indicated concentrations of EGF (Peprotech Cat# AF-100-15), recombinant human AREG (Peprotech Cat# 100-55B), osimertinib (Selleck Chemical LLC, Houston, TX, USA, Cat# S7297), or necitumumab (Selleck Chemical LLC Cat# A2048) were included in the cell media. CTG assays were performed in triplicate at 0 h and 72 h, and normalized to 0 h DMSO-treated control. In experiments using osimertinib and necitumumab, all cells received the same final volume of the drug vehicle DMSO. In the combination drug experiments, concentrations were selected based on approximate IC_50_s of osimertinib and necitumumab.

### 2.3. Immunoblot Assays

For EGFR expression immunoblots, MCF10A cells stably transduced with empty vector, EGFR WT, EGFR N361A, EGFR L858R, or EGFR N361A/L858R were stimulated for 15 min with an additional 20 ng/mL EGF. For drug treatment immunoblots, MCF10A cells stably transduced with empty vector, EGFR WT, or EGFR N361A were treated with 10 µM necitumumab or 100 nM osimertinib for 1 h. Cells were harvested from tissue culture plates, centrifuged for 300× *g* for 5 min, washed 2 times with 1× ice-cold PBS, and then resuspended into 3 volumes of ice-cold RIPA (Thermo Fisher Scientific Cat# 89901) and 1× HALT buffer (Thermo Fisher Scientific Cat# 78445). Lysates were incubated on ice for 30 min with gentle vortexing every 5 min, centrifuged at 14,000× *g* for 10 min at 4 °C in an Eppendorf 5427R tabletop centrifuge. Relative protein concentrations in each supernatant were quantified with a Pierce BCA assay kit (Thermo Fisher Scientific Cat# 23225) and Varioskan Lux. Samples were boiled at 70 °C for 10 min with a final concentration of 50 mM dithiothreitol (Thermo Scientific Cat# R0861) to prevent disulfide bridge formation. Samples were then electrophoresed on NuPAGE™ 4–12% Bis-Tris 1.5 mm Mini Protein Gels (Invitrogen Cat# NP0322BOX), and transfers were performed using an iBlot2 (Thermo Fisher Scientific Cat# IB21001) with mini nitrocellulose stacks (Invitrogen Cat# IB23002).

Subcellular fractionation was performed using the Mem-PER Plus Membrane Protein Extraction Kit (Thermo Fisher Cat# 89842). As a modification to the standard protocol provided by this kit, 1× HALT Protease and Phosphatase Inhibitor Cocktail (Thermo Scientific Cat#87785) was added to both the solubilization and permeabilization buffers.

Membranes were probed at 4 °C overnight with the following primary antibodies in 3% bovine serum albumin (BSA) (Sigma-Aldrich Cat# A7906) in 1× TBS-0.1% Tween-20: 1:5000 β-Actin (Sigma-Aldrich Cat# A5441, RRID:AB_476744), 1:500 phospho-EGFR Y1068 (Cell Signaling Technology Cat# 2236, RRID:AB_331792), 1:500 phospho-ERK1/2 T202/Y204 (Thermo Fisher Scientific Cat# 14-9109-82, RRID:AB_2572926), 1:500 phospho-AKT S473 (Cell Signaling Technology, Danvers, MA, USA, Cat# 4060, RRID:AB_2315049), 1:1000 GADPH (Cell Signaling Technology Cat# 5174, RRID:AB_10622025), 1:500 EGFR (Cell Signaling Technology Cat# 2232, RRID:AB_331707), and 1:1000 ATP1A1 (Abcam, Cambridge, UK, Cat# ab76020, RRID:AB_1310695). The secondary antibodies used were 1:10,000 goat anti-mouse IRDye® 800CW (LI-COR Biosciences Cat# 92632210, RRID:AB_621842) and 1:10,000 goat anti-rabbit IRDye® 680RD (LI-COR Biosciences Cat# 92668071, RRID:AB_10956166). Image processing was performed by a LI-COR Odyssey CLx (LI-COR Biosciences, Lincoln, NE, USA, Cat# 9140-09) or ChemiDoc MP (Bio-Rad, Hercules, CA, USA, Cat# 12003154) machine and Image Studio (version 5.2.5) and Image Lab (version 6.1) software from LI-COR Biosciences and Bio-Rad, respectively. Full uncropped immunoblot images are available in Figure S7.

### 2.4. Fluorescent Microscopy Assays

Live cells were plated at 200,000 cells/well in either clear-bottom 12-well plates (Corning, Corning, NY, USA, Cat# 353043) with poly-L-lysine-coated 18 mm coverslips (neuVitro, Camas, WA, USA, Cat# GG-18-1.5-PLL) or glass-bottom 12-well plates (MatTek Corp., Ashland, MA, USA, Cat# P12G-1.5-10-F). The next day, the cells were briefly washed with ice-cold 1× PBS (R&D Systems Cat# 4870-500). Cells were fixed in 4% formaldehyde (Thermo Scientific Chemicals Cat# J60401) for 15 min at room temperature, then washed three times with 1× PBS at room temperature for 5 min on an orbital shaker at 35 revolutions per minute (RPM). Cells were permeabilized with 0.1% Triton X-100 (Fisher Chemicals, Pittsburgh, PA, USA, Cat# BP151-500) in 1× PBS for 10 min at room temperature, and then washed three times with 1× PBS for 5 min on an orbital shaker. Wells were blocked with Rockland Blocking Buffer (Rockland Immunochemicals, Limerick, PA, USA, Cat# MB070) for 30 min and probed as indicated with the following primary antibodies in 1% BSA in 1× PBS-0.1% Tween-20: 1:50 Alexa-Fluor 594-conjugated Vimentin (Cell Signaling Technology Cat# 7675, RRID:AB_ 2797632), Alexa-Fluor 647-conjugated ATP1A1 (Invitrogen Cat# MA3-928-A647, RRID:AB_2633350), and 1:100 Alexa Fluor 488-conjugated EGFR (Cell Signaling Technology Cat# 5616, RRID:AB_10691853). Coverslips were incubated in antibody dilution in a humidified chamber at 4 °C overnight at minimum oscillation speed in a light-blocking container. Antibody mixtures were aspirated, and then cells were washed three times with 1× PBS for 5 min at room temperature on an orbital shaker in a light-blocking container. Coverslips were counterstained by adding mounting media with 4′,6-diamidino-2-phenylindole (DAPI) (Vectashield, Newark, CA, USA, Cat# H-1000-10), and then placed over the DAPI drop on the microscope slide and pressed gently to remove bubbles and dabbed to remove excess media if necessary, before sealing. Imaging was performed using the Nikon A1 Confocal microscope (Nikon, Melville, NY, USA) with 60× objective oil immersion. Image processing was performed using the NIS Elements software (version 4.6).

### 2.5. Flow Cytometry

After trypsinization and neutralization with normal media, cells were filtered through 35 µm cell strainer caps (Corning Cat# 352235). A subset of cells were diluted 1:1 in Trypan blue (Invitrogen Cat# T10282), and live cells were quantified using Countess Cell Counting Slides (Invitrogen Cat# C10228) on a Countess 3 Automated Cell Counter (Invitrogen Cat# AMQAX2000). Approximately 500,000 cells were fixed and permeabilized by 4% formaldehyde (Thermo Fisher Scientific Cat# J60401-AP) and 90% ice-cold methanol (Sigma-Aldrich Cat# 34860), respectively. Cells were then incubated with 1:50 Alexa-Fluor 488-linked EGFR (Cell Signaling Technology Cat# 5616, RRID:AB_10691853) in flow buffer for 1 h at room temperature in a light-blocking container. Unbound antibodies were washed out, and cells were resuspended in fresh flow buffer.

To measure EGFR expression after transduction, flow cytometry was performed on a BD FACSCalibur flow cytometer. Live cells were gated by side scatter and forward scatter. Alexa-Fluor 488-conjugated EGFR-positive cells were gated using parameters in which unstained controls had <0.1% positive cells. The Alexa-Fluor 488-linked EGFR antibody (Cell Signaling Technology Cat# 5616, RRID:AB_10691853) used for flow cytometry was selected because it binds the intracellular domain, minimizing the chances that N361A impacts binding.

For EGF binding experiments, MCF10A cells stably expressing EGFR WT or EGFR N361A were serum-starved overnight, then were treated with 100 nM EGF-fluorescein conjugate (Invitrogen Cat #E3478) for 1 h on ice in the dark. Parental cells were used as a control, rather than empty vector, because the empty vector (RRID:Addgene_14430) expresses eGFP. Flow cytometry was performed on a Beckman Coulter CytoFLEX flow cytometer (Indianapolis, IN, USA). Live cells were gated by side scatter and forward scatter.

### 2.6. In Situ Proximity Ligation Assay

The protocol for proximity ligation assays (PLA) was performed as previously described [37]. For each cell line, live cells were counted using a Countess 3 Automated Cell Counter and seeded at 200,000 cells per well in glass-bottom 12-well plates (MatTek Corp. Cat# P12G-1.5-10-F) in their respective complete media. The following day, MCF10A cells were subsequently washed with ice-cold 1× PBS (R&D Systems Cat# 4870-500) three times. 293T cells were incubated with an additional 20 ng/mL EGF for 15 min, then subsequently washed with ice-cold 1× PBS three times.

The DUOLINK in situ Far-Red kit (Sigma-Aldrich Cat# DUO92013) was used for the PLA. Wells were blocked in accordance with the manufacturer’s protocol, then probed with the following primary antibodies in DUOLINK Antibody Diluent and its corresponding dilutions: 1:100 anti-rabbit monoclonal HER2/ErbB2 (Cell Signaling Technology Cat# 2165, RRID:AB_10692490) and 1:100 anti-mouse monoclonal EGFR (Genetex, Sinchu City, Taiwan, Cat# GTX628887, RRID:AB_2888064). To avoid possible interactions with the extracellular N361 region, these antibodies each bind the intracellular domain of their target: residues surrounding Tyr1248 of HER2 and the C-terminal region of human EGFR. Well plates were incubated in primary antibody dilution as mentioned above in a humidified chamber at 4 °C overnight at minimum oscillation speed in a light-blocking container. Wells were probed with DUOLINK PLA probe solution, using equal amounts of PLA probe 1:5 Donkey anti-Mouse MINUS (Sigma-Aldrich Cat# DUO92004, RRID:AB_2713942) and 1:5 Donkey anti-Rabbit PLUS (Sigma-Aldrich Cat# DUO92002, RRID:AB_2810940) diluted in DUOLINK Antibody diluent for an hour in a humidified chamber at 37 °C. Ligation was then performed by incubating wells with DUOLINK ligation solution (1:40 1 U/µL ligase in 1× ligation buffer in high-purity H_2_O), followed by DUOLINK amplification solution (1:80 10 U/µL polymerase in 1× amplification FarRed buffer in high-purity H_2_O). All incubation steps at 37 °C were performed without any shaking or oscillation, and incubation steps at 4 °C were performed on an orbital shaker at 35 revolutions per minute (RPM). Each well was then incubated with two drops per mL of ActinRed 555 ReadyProbe fluorescence dye (Sigma-Aldrich Cat# R37112), and then washed twice with 1× PBS at room temperature on an orbital shaker at 35 RPM. Finally, wells were counterstained with DUOLINK in situ mounting media with DAPI (Sigma-Aldrich Cat# DUO82040) and incubated at room temperature for 15 min.

Imaging was performed using the Nikon A1 Confocal microscope with 60× objective oil immersion. Image processing was performed using the included NIS Elements software (version 4.6). Cell boundaries were defined using ActinRed channel. Quantification of mean pixel intensity per cell line was determined by CellProfiler Image Analysis Software (version 4.2.7, RRID:SCR_007358) [38]. The same settings were applied to each of the 5 replicates per condition.

### 2.7. Statistical Analyses

Statistical tests are described in figure legends, and all other tests were two-tailed T-tests performed in GraphPad Prism 10 (RRID:SCR_002798) or Microsoft Excel (version 16.105.1, RRID:SCR_016137). Power analysis was performed to determine the number of replicates needed for the significance level of p<0.05. For time- and dose-course experiments, Varioskan data were processed using SkanIt. (Themo Fisher Scientific, version 7.0) Two-way analysis of variance (ANOVA), followed by Dunnett’s multiple comparison test, was used to analyze time- and dose-course experiments. Inhibitor treatment data were normalized to control vehicle-treated conditions at day 3, and then normalized again to cell numbers at day 0. For each condition, exponential growth equations, outlier analysis, and area under the curve were calculated using GraphPad Prism 10. Flow cytometry analysis was performed using Flowing Software 2.5.1 (Turku Bioscience, Turku, Finland), CellQuest Pro (version 5.2.1) (BD Biosciences, San Jose, CA, USA), and CytExpert software (version 2.6). One-way analysis of variance (ANOVA), followed by Tukey’s multiple comparisons test, was performed to analyze direct-stain flow cytometry. To analyze images from EGFR-HER2 in situ proximity ligation assay coupled with fluorescence microscopy, a Kruskal–Wallis test, followed by Dunn’s multiple comparisons test, was performed. Normalized mean intensity was calculated using CellProfiler Image Analysis Software (RRID:SCR_007358) employing the MeasureObjectIntensity module, which scales pixel intensity within each cell in the image from 0 to 1. For all figures, *p*-values are shown as follows: ns represents not significant, * *p* < 0.05, ** *p* < 0.01, *** *p* < 0.001, **** *p* < 0.0001. Data were plotted in box-and-violin arrangement in GraphPad Prism 10. Figures were created in Adobe Illustrator (version 30.1).

## 3. Results

### 3.1. Stable Overexpression of Mutant EGFR N361A

**Figure 1:**
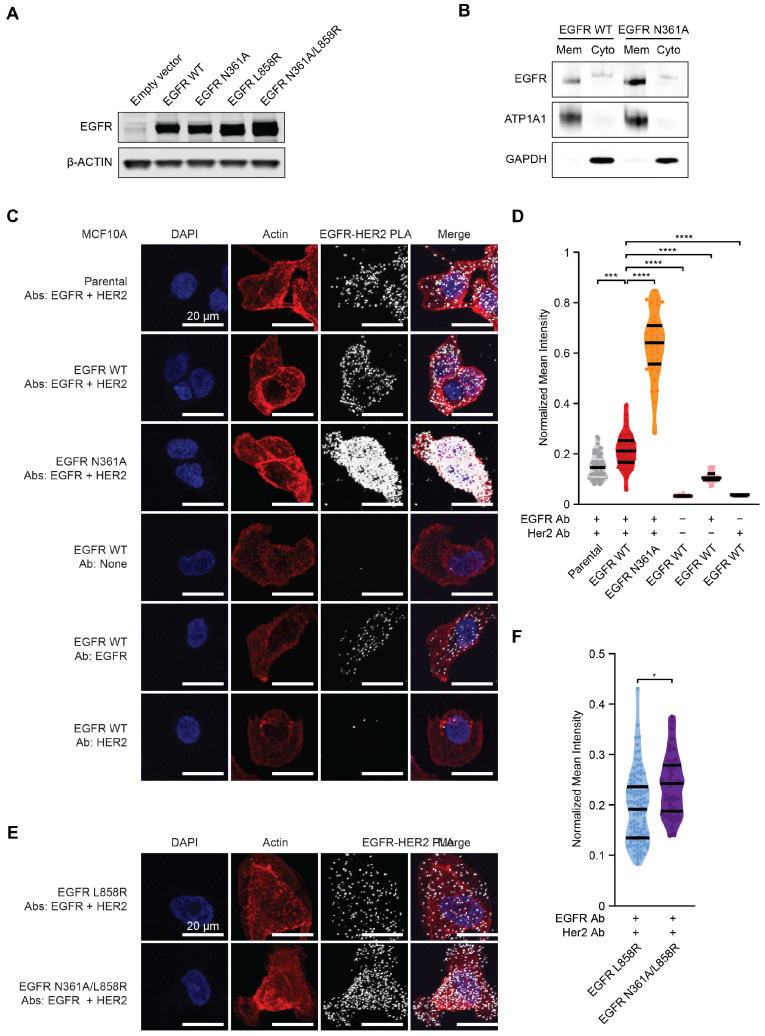
EGFR N361A induces increased co-localization of EGFR and HER2. (**A**) Immunoblots of whole cell lysates of parental MCF10A cells or MCF10A cells overexpressing cDNAs of EGFR WT, EGFR N361A, EGFR L858R, or EGFR N361A/L858R. Lysates were probed as indicated. (**B**) Subcellular fractionation of membrane-enriched (Mem) and cytosol-enriched (Cyto) fractions was performed on MCF10A cells stably overexpressing EGFR WT or EGFR N361A. The membrane loading control was Sodium Potassium-Transporting Subunit Alpha-1 (ATP1A1), and the cytosolic loading control was GAPDH. (**C**) Fluorescent microscopy images of MCF10A cells that were parental, overexpressed exogenous EGFR WT, or overexpressed exogenous EGFR N361A. In situ proximity ligation assays used the indicated primary antibodies against EGFR and/or HER2. Blue: DAPI; Red: Rhodamine Actin; White: EGFR-HER2 PLA. Scale bar represents 50 µm. For lower magnification images, please see Figure S4A. (**D**) Quantification of mean PLA pixel intensity within actin-defined cellular boundaries. (**E**) Fluorescent microscopy images of MCF10A cells that overexpressed exogenous EGFR L858R or a single construct containing two mutations EGFR N361A/L858R, using primary antibodies against EGFR and HER2. Blue: DAPI; Red: Rhodamine Actin; White: EGFR-HER2 PLA. Scale bars (red) represent 20 µm. For lower magnification images, please see Figure S4B. (**F**) Quantification of mean PLA pixel intensity within actin-defined cellular boundaries. For all panels, n = 5 images and * p<0.05, *** p<0.001, **** p<0.0001. Uncropped western blots are presented in Figure S7.

To study the effects of glycosylation at N361 of EGFR, site-directed mutagenesis was performed on EGFR overexpression plasmids to create the glycosylation-deficient mutant EGFR N361A. This was performed using either full-length wild-type EGFR cDNA or EGFR cDNA containing the oncogenic L858R mutation. We transduced non-transformed MCF10A and 293T cells, as these model systems do not have hyperactivation of the EGFR/KRAS pathway by pre-existing cancer-inducing mutations, and then selected the cells for stable EGFR overexpression. MCF10A cells normally proliferate in 20 ng/mL of EGF and express endogenous EGFR, and as a result, they are reliant on this signaling pathway. Compared with MCF10A cells, 293T cells express less endogenous EGFR, and their media is not typically supplemented with EGF (Figure S1). We stably transduced MCF10A and 293T cells with similar levels of wild-type EGFR or EGFR N361A and selected the constructs using puromycin (Figure S2A,B). Immunoblots, flow cytometry, and fluorescent microscopy of MCF10A and 293T cells showed that stable overexpression of EGFR constructs were relatively similar and that all constructs were significantly higher than endogenous EGFR in empty vector or non-transduced parental cells (Figure 1A and Figure S2C–J). Immunofluorescent microscopy experiments demonstrated that mutant EGFR N361A construct was expressed and was localized to the cytoplasm and cell membrane (Figure S3). In cell fractionation experiments using MCF10A cells, both EGFR WT and EGFR N361A were predominantly localized to the membrane fraction; however, EGFR N361A exhibited higher overall membrane enrichment compared with EGFR WT (Figure 1B).

### 3.2. Co-Localization of EGFR and HER2

Glycosylation can regulate the heterodimerization of receptor tyrosine kinases [39]. To evaluate the impact of the glycosylation-deficient N361A mutant on the co-localization of EGFR and its binding partner HER2, we performed proximity ligation assays (PLA) on the MCF10A cell line panel after 15 min of stimulation with the agonist ligand EGF [40]. EGF promotes dimerization between EGFR and HER2 [41]. Interestingly, EGFR N361A cells had threefold elevated PLA intensity per cell relative to EGFR wild-type or negative unstained controls, indicating that N361A drove increased co-localization (Figure 1C,D and Figure S4A). Concordantly, the EGFR N361A/L858R double mutant displayed significantly more co-localization with HER2 than L858R alone (Figure 1E,F and Figure S4B). While co-localization in the glycosylation-deficient mutant increased, it was unclear from the PLA assay alone whether the co-localization represents productive dimerization resulting in signaling. Increased membrane localization of the N361A mutant may account for the increased co-localization between EGFR and HER2 observed in the mutant context (Figure 1B–F).

### 3.3. Differential Effects of EGFR N361A on Proliferation in Response to Ligand Stimulation

**Figure 2:**
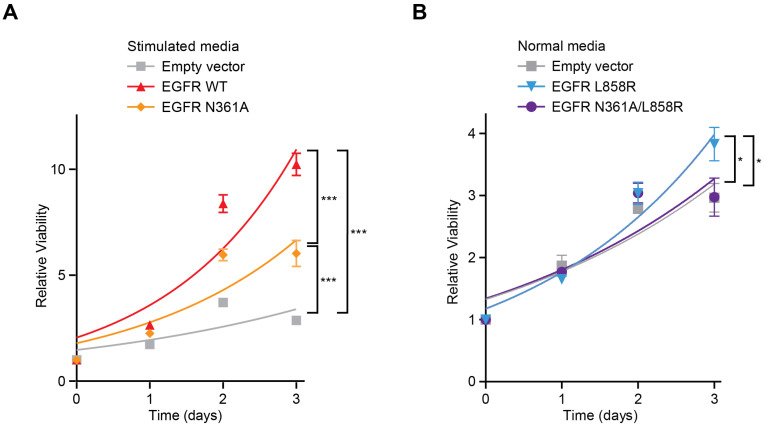
EGFR N361A suppresses proliferation relative to wild-type EGFR. (**A**) Time course of relative viability by CellTiter-Glo (CTG) of MCF10A cells expressing control empty vector (grey), cDNAs of wild-type EGFR (red), or cDNA of EGFR N361A (orange) in media supplemented with an additional 20 ng/mL EGF. (**B**) Time course of relative viability by CTG of MCF10A cells overexpressing cDNAs of empty vector control (grey), EGFR L858R (blue), or a single construct containing two mutations EGFR N361A/L858R (purple) in normal MCF10A media. For all panels, n = 3, error bars represent SEM, * p<0.05, *** p<0.001.

To determine the effects of glycosylation at N361 on cell proliferative responses, including responses to natural agonist ligands, we compared the proliferation of our stably transduced cell lines across 3-day time courses. Overexpression of EGFR WT in MCF10A cells in EGF-stimulated conditions led to accelerated cell proliferation relative to empty vector (Figure 2A). However, overexpression of EGFR N361A caused a significantly smaller increase in proliferation (Figure 2A). In normal media, cells with double mutant EGFR N361A/L858R proliferated less than L858R alone (Figure 2B). Thus, removing glycosylation at N361 caused proliferative deficiency.

**Figure 3:**
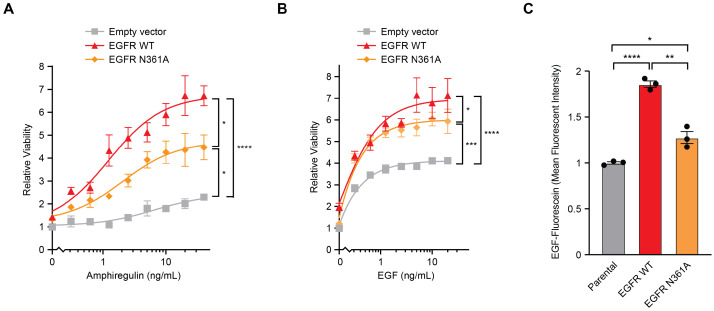
EGFR N361A decreases responses to growth factors relative to wild-type EGFR. (**A**,**B**) Dose course of relative viability by CTG of MCF10A cells expressing control empty vector (grey), cDNAs of wild-type EGFR (red), or EGFR N361A (orange) grown in the presence of increasing doses of the ligand AREG (**A**) or EGF (**B**) for 72 h. The zigzag represents a discontinuous axis. Data normalized to day 0 empty vector untreated. (**C**) Mean fluorescence intensity (MFI) of live MCF10A cells stably transduced with plasmids containing EGFR WT or EGFR N361A, treated with 100 nM EGF-Fluorescein for 1 h and assessed by flow cytometry, with MFI normalized to parental cells. For all panels, n = 3, error bars represent SEM, * p<0.05, ** p<0.01, *** p<0.001, **** p<0.0001.

We next tested whether the N361A mutation affected the ability of EGFR to respond to proliferation-stimulating ligand signals. In a 72 h dose course of AREG, at high AREG concentrations, EGFR WT increased proliferation relative to empty vector significantly more than EGFR N361A (Figure 3A). Similarly, cells with EGFR N361A were deficient at converting higher concentrations of EGF into increased proliferation relative to cells with EGFR WT (Figure 3B). To further assess how N361A alters EGF binding, these cells were treated with EGF conjugated to fluorescein, and binding was assessed by flow cytometry. Overexpression of EGFR N361A led to significantly less EGF binding relative to EGFR WT (Figure 3C and Figure S5). These results show that N361A impaired EGF binding and decreased proliferative response to ligands.

### 3.4. N361A Desensitizes Cells to Inhibition of EGFR with Extracellular Antibody Necitumumab

**Figure 4:**
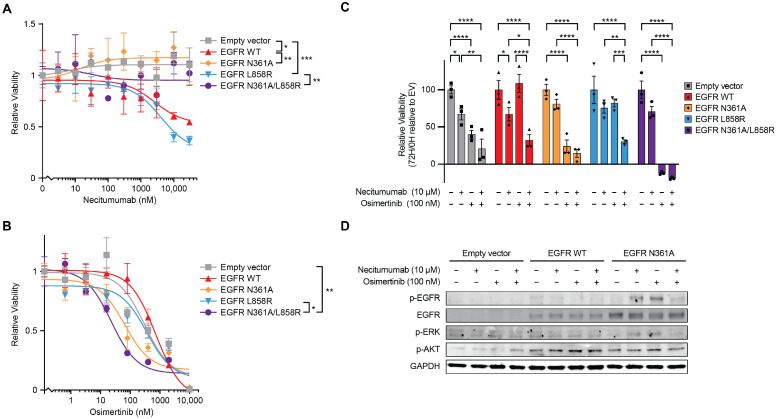
EGFR N361A causes resistance to necitumumab and sensitization to osimertinib. (**A**) Dose course of relative viability measured by CTG after 72 h of treatment with necitumumab of either parental MCF10A cells or MCF10A cells overexpressing cDNAs of EGFR WT, EGFR N361A, EGFR L858R, or EGFR N361A/L858R. (**B**) Dose course of relative viability measured by CTG after 72 h of treatment with osimertinib of either parental MCF10A cells or MCF10A cells overexpressing cDNAs of EGFR WT, EGFR N361A, EGFR L858R, or EGFR N361A/L858R. (**C**) Combination antagonist assay of relative viability measured by CTG of MCF10A cells stably expressing the indicated constructs and treated with necitumumab (10 µM), osimertinib (100 nM), both, or an equal dose of negative control DMSO for 72 h. The zigzag represents a discontinuous axis. For all panels, n = 3, error bars represent SEM, * p<0.05, ** p<0.01, *** p<0.001, **** p<0.0001. (**D**) Immunoblot on lysates from MCF10A cells stably expressing empty vector, EGFR WT, or EGFR N361A treated with DMSO, 10 µM necitumumab, 100 nM osimertinib, or both compounds for 1 h, and probed as indicated. Uncropped western blots are presented in Figure S7.

To assess the impact of N361A on antibody inhibitors of the extracellular domain of EGFR, we used necitumumab, a clinically approved antibody inhibitor that targets domain III of EGFR [14]. Ten-point necitumumab dose course treatments from 0 to 30 µM demonstrated that necitumumab significantly inhibited proliferation of MCF10A cells expressing wild-type EGFR cDNA (IC_50_ of 2.6 µM) (Figure 4A). However, the cell line expressing EGFR N361A was completely insensitive to the antibody inhibitor, much like the vector control (Figure 4A). This change was even more pronounced in MCF10A cells overexpressing EGFR L858R, which were highly sensitive to necitumumab (MCF10A IC_50_ 4.0 µM), when compared with the lines overexpressing the double mutant N361A/L858R, which were completely insensitive to necitumumab (Figure 4A). We observed similar results in 293T cells (293T IC_50_ 4.5 µM), although those were more sensitized to necitumumab by EGFR L858R than by EGFR WT (Figure S6A). MCF10A cells express more endogenous EGFR than 293T cells, and MCF10A cells are normally grown in EGF, both of which may make MCF10A cells more susceptible to addiction to EGFR signaling (Figure S1) [42].

Knowing that the drug response of EGFR may be dependent on its glycosylation state, we evaluated the response of cells expressing EGFR N361A to the tyrosine kinase domain inhibitor osimertinib, which is used clinically against NSCLC with EGFR L858R mutations or exon 19 deletions [12,43]. MCF10A cells stably expressing the double mutant N361A/L858R were significantly more sensitive to osimertinib than the single mutant L858R cells (N361A/L858R IC_50_ 22.8 nM, L858R IC_50_ 361.4 nM) (Figure 4B). This difference was not observed in 293T cells, which were equally insensitive to osimertinib at all but the highest of doses (Figure S6B). These results further emphasize the importance of considering the EGFR glycosylation state when deciding on a treatment course for cancer patients.

Combination therapies of anti-EGFR antibodies with small molecules that block EGFR tyrosine kinase activity have shown benefit to patients with NSCLC [44]. In MCF10A cells expressing EGFR WT or EGFR L858R, the addition of necitumumab increased the efficacy of osimertinib relative to osimertinib alone (Figure 4C). Strikingly, in N361A and N361A/L858R cells, the addition of necitumumab to osimertinib did not show a significant additive effect relative to osimertinib alone. This is consistent with a model where glycosylation at N361 is required for necitumumab binding. In EGFR WT cells, treatment with necitumumab and/or osimertinib slightly reduced p-EGFR and p-ERK (Figure 4D). In contrast, in N361A cells, treatment with EGFR inhibitors induced p-EGFR and p-ERK (Figure 4D). Phosphorylation of AKT was induced by the overexpression of EGFR WT and N361A; however, it was unaffected by EGFR inhibitors. The N361A mutant blocked the ability of inhibitors to reduce p-EGFR and p-ERK and prevent proliferation, suggesting that the glycosylation site N361 is required for necitumumab to be effective.

## 4. Discussion

Collectively, our data on proliferation, co-localization, and signaling in MCF10A cells suggest a model where the glycosylation of EGFR at N361 helps EGFR convert growth factors into proliferative signals. Disruption of this glycosylation site led to a moderate increase in the localization of EGFR to the membrane and the formation of dominant non-functional co-localization events between EGFR and its canonical binding partner HER2 in MCF10A cells [45,46]. Dimerization and activation of EGFR can occur as separate events with different thresholds, and inactive dimers that do not induce kinase activity can form [45,46]. Previous recombinant studies using in vitro purified soluble mutant EGFR N361E demonstrated that this mutant was able to dimerize in response to EGF [47]. HER2 heterodimerization with EGFR induces structural and phosphorylation changes in the carboxy tail, which can be impacted by non-functional dimerization [41,45,46].

Structurally, N361 is located in the extracellular domain of EGFR proximal to the EGF binding site, which is formed from the cavity of Domains I through III [30]. Both EGF-ligand binding and N-glycosylation of EGFR stabilize and place Domain III monomers closer together [39]. In molecular dynamic simulations, glycosylation groups on EGFR N361 were predicted to bind to EGFR residues 329–333, which are directly involved in electrostatic binding to dimerization partners [21]. Glycosylation at N361 may impact the structure of this domain, leading to an increased affinity for EGF. Consistent with a model where N361A mutation negatively impacts EGF binding affinity, the alanine mutant at N361 reduced EGF binding by flow cytometry and resulted in a decreased efficacy of EGF on promoting the proliferation of MCF10A cells relative to wild-type. The double mutant of N361A and L858R was less sensitive to EGF than the single L858R mutant at low concentrations in MCF10A cells. Glycosylation at N361 may shift the receptor L domain of EGFR into a conformation with a higher affinity for ligand binding.

ErbB receptors may be tuned to be more oncogenic by the upregulation of N-glycan groups [48,49]. Other glycosylation sites such as N151, N328, N444, and N603 on EGFR, and the choice of ligand, may impact how the ligand binding domain acts to stabilize EGFR-EGF binding [21,32]. Mutation of the glycosylation site at EGFR N444 has been implicated in a EGF binding deficiency and a defective autophosphorylation of the intracellular domain in response to ligands [47,50,51]. N444E stimulated a ligand-independent change in tyrosine autophosphorylation, while N361E required EGF for tyrosine phosphorylation to be induced [47]. Relative to wild-type EGFR, unglycosylated EGFR N603 mutants demonstrated increased propensity to dimerize without ligand, but were deficient at increasing cell survival in the absence of stimulatory ligand [25]. Under normal conditions, mutation at N361A caused a slight decrease in the phosphorylation of EGFR and ERK in MCF10A cells. EGFR inhibitors paradoxically induced EGFR signaling in the EGFR N361A mutant cells more than in the EGFR WT cells, suggesting that, in cases of exogenous inhibitor treatment, the increased co-localization may drive productive signaling. This surprising result, which suggests that glycosylation at N361 may be important for drug sensitivity in MCF10A cells, warrants further study in additional systems. EGF has higher affinity binding for EGFR than AREG, and as expected, stimulation with EGF led to larger increases in proliferation than AREG [52,53]. EGF preferentially binds HER2/EGFR heterodimers compared with EGFR homodimers, while AREG has no preference and is less efficient at stimulating homodimerization than EGF [54].

Consistent with this study’s results that the glycosylation of EGFR influences responses to anti-EGFR therapeutic agents, molecular dynamics analyses of co-crystal structures of EGFR with inhibitory antibody mAb806 found that glycosylation can, in some instances, increase the exposure of the bound epitope [22]. Mutating away the glycosylation site by introducing N361A may change the EGFR ectodomain structure so that single-agent necitumumab treatment is not able to bind effectively. In contrast, decreasing EGFR glycosylation causes cells to be sensitized to kinase domain inhibitors like osimertinib [55]. In ovarian, colorectal, and pancreatic cancer cell lines, overexpression of a glycosylation-promoting enzyme, ST6Gal-I sialyltransferase, increased EGFR activation and enhanced resistance to cell death caused by treatment with an inhibitor of EGFR kinase activity, gefitinib [55]. The increased EGFR signaling promoted by glycosylation may decrease the efficacy of EGFR kinase domain inhibitors. Resistance to EGFR inhibitors can be induced by activating mutations of downstream effectors of the signaling pathway, such as KRAS [56,57]. KRAS mutations can drive aberrant glycosylation in pancreatic ductal adenocarcinoma [58]. CRISPR screens for genes that were synthetically lethal with KRAS inhibition identified multiple genes regulating glycosylation, suggesting that glycosylation is important for the efficacy of inhibiting this pathway [59].

Glycosylation is frequently elevated in tumor cells, and inhibitors of glycosylation are tools that can be used to further explore the roles of glycosylation in cancer [60]. Tunicamycin inhibits UDP-GlcNAc-1-phosphotransferase, an enzyme critical for N-glycosylation [61]. It can decrease EGFR glycosylation, leading to instability, reduced activating EGFR phosphorylation, and cancer cell-selective decreases in ERK signaling [62]. Glycosylation subtypes like sialic acid-binding immunoglobulin-like lectin 7 (Siglec-7) can promote immune evasion, motivating the development of Siglec inhibitors [63]. Lung cancer cell lines with genomic alterations in EGFR tended to be among the most highly sensitive to the glycosylation inhibitor NGI-1, which can be paired with EGFR inhibitors to overcome acquired resistance [64]. Another promising strategy to treat tumors driven by overactive EGFR signaling is to target upstream enzymes required for glycosylation, such as GOLPH3 or UGP2 [27,28,65].

## 5. Conclusions

In this study, the ablation of the glycosylation site at N361 of EGFR led to increased localization to the cellular membrane, increased co-localization with HER2, decreased proliferation over time, and decreased binding and sensitivity to ligands. This data is consistent with a model of N361A inducing dominant non-functional colocalization events that inhibit downstream functions. This study’s findings that the glycosylation-deficient mutant N361A improved the efficacy of the EGFR tyrosine kinase inhibitor osimertinib in MCF10A cells suggest that strategies decreasing the glycosylation of EGFR may have clinical relevance at improving the efficacy of the EGFR tyrosine kinase inhibitors. The present study was performed using overexpression systems without pre-existing activating mutations in EGFR or downstream members of the pathway. Future studies of EGFR N361A in models of non-small cell lung cancer, particularly using CRISPR at the endogenous genomic locus, would expand understanding of this site’s importance in cancer systems and provide an excellent model for further glycosylation studies. Thus, understanding the roles of protein glycosylation can lead to new avenues of exploring cell biology and improving cancer therapy.

## Data Availability

The data and reagents from the study are available in the article and the Supplementary Files. Plasmids generated in the study are available from Addgene. A preprint of this article is available on bioRxiv (doi: https://doi.org/10.1101/2024.07.12.603279).

## Supplementary Materials

The following supporting information can be downloaded at https://www.mdpi.com/article/10.3390/cancers18030474/s1: Figure S1: EGFR expression; Figure S2: EGFR overexpression constructs in cells; Figure S3: Immunofluorescence of EGFR mutants; Figure S4: Proximity ligation assays from Figure 1B,D; Figure S5: Flow cytometry data and gating corresponding to Figure 3C; Figure S6: EGFR mutants modulate the effectiveness of EGFR inhibitors; Figure S7: Uncropped immunoblot images for Figure 1A,B and Figure 4D.
