# Supplementary material for: Effects of N361 Glycosylation on Epidermal Growth Factor Receptor Biological Function
Source: Cancers (Basel). 2026 Jan 31;18(3):474. doi: 10.3390/cancers18030474 (PMC12896819; doi:10.3390/cancers18030474)
Supplement: Supplementary file 1 [file cancers-18-00474-s001.zip › cancers-4086028-supplementary.pdf]

Effects of N361 Glycosylation on Epidermal Growth Factor Receptor Biological Function

Dennis Lam, Brandon Arroyo, Ariel N. Liberchuk, Jessica Das, Leonard J. Ash, Khizr M. Khan, Jayati Mondal, and Andrew L. Wolfe

Supplementary Materials

This file includes Supplementary Figure S1, Figure S2, Figure S3, Figure S4, Figure S5, Figure S6, and Figure S7.

Figure S1

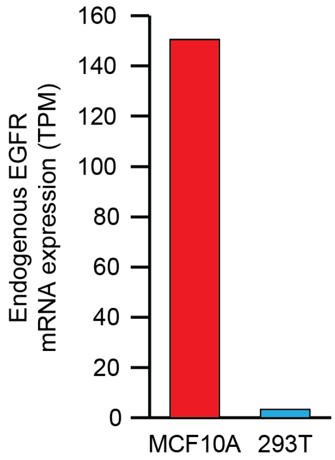

Figure S1. EGFR expression. mRNA expression of EGFR in MCF10A and HEK-293T cells from single-cell RNAseq data. Adapted from the Human Protein Atlas.

Figure S2

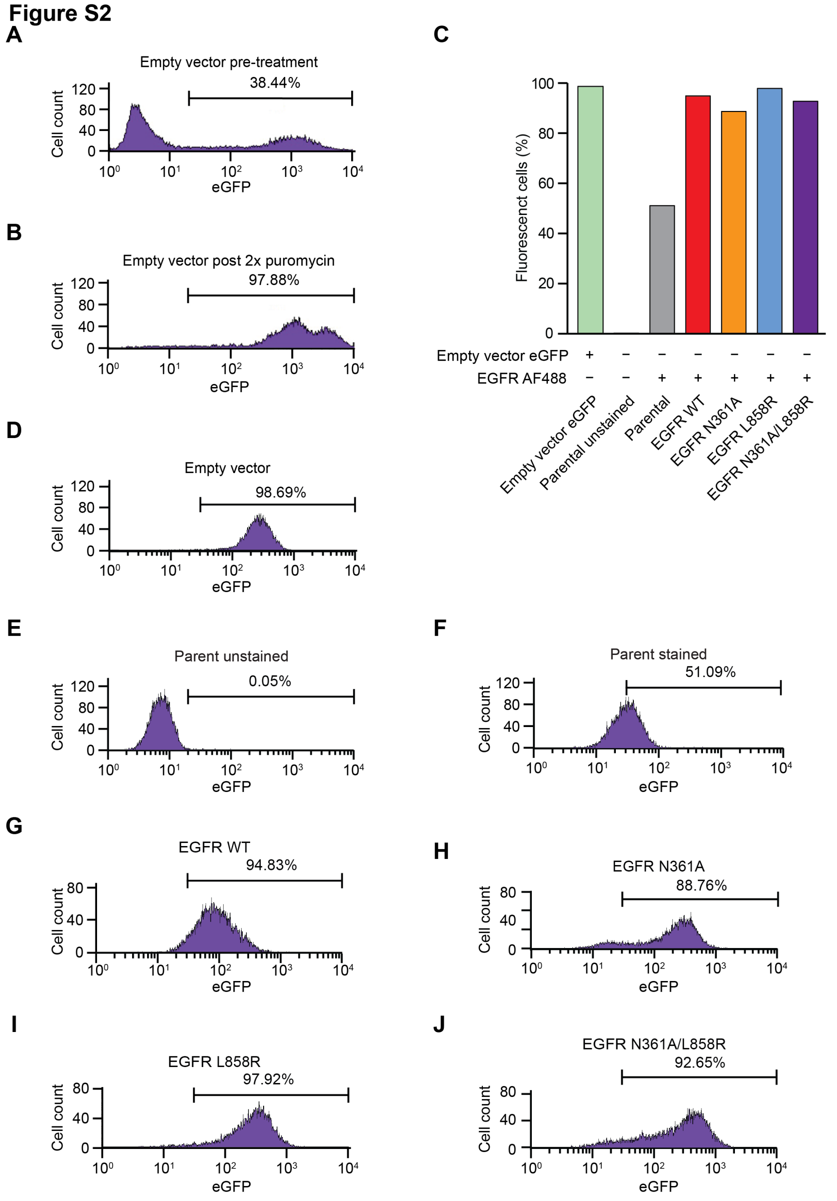

Figure S2. EGFR overexpression constructs in cells. (A-B), Histogram of flow cytometry data showing eGFP in positive control MCF10A cells overexpressing fluorescent puro-IRES-eGFP plasmid (A) before and (B) after two rounds of puromycin selection. **(C),** Flow cytometry results showing the percentage of fluorescence positive gated MCF10A cells, either parental or overexpressing wild-type EGFR (EGFR WT), EGFR N361A, EGFR L858R, or a single construct containing two mutations EGFR N361A/L858R. Cells were labeled with stable empty fluorescent vector puro-IRES-GFP or an anti-EGFR antibody conjugated to Alexa-Fluor 488 as indicated. **(D-J)**, Histograms of flow cytometry data of cells stained with a fluorescent EGFR-AF488 antibody or control unstained cells. Shown are (D) unstained parental MCF10A cells expressing puro-IRES-eGFP, (E) unstained parental cells, (F) stained parental cells, (g) EGFR WT cDNA, (H) EGFR N361A cDNA, (I) EGFR L858R cDNA, or (J) a single cDNA construct containing two mutations EGFR N361A/L858R.

Figure S3

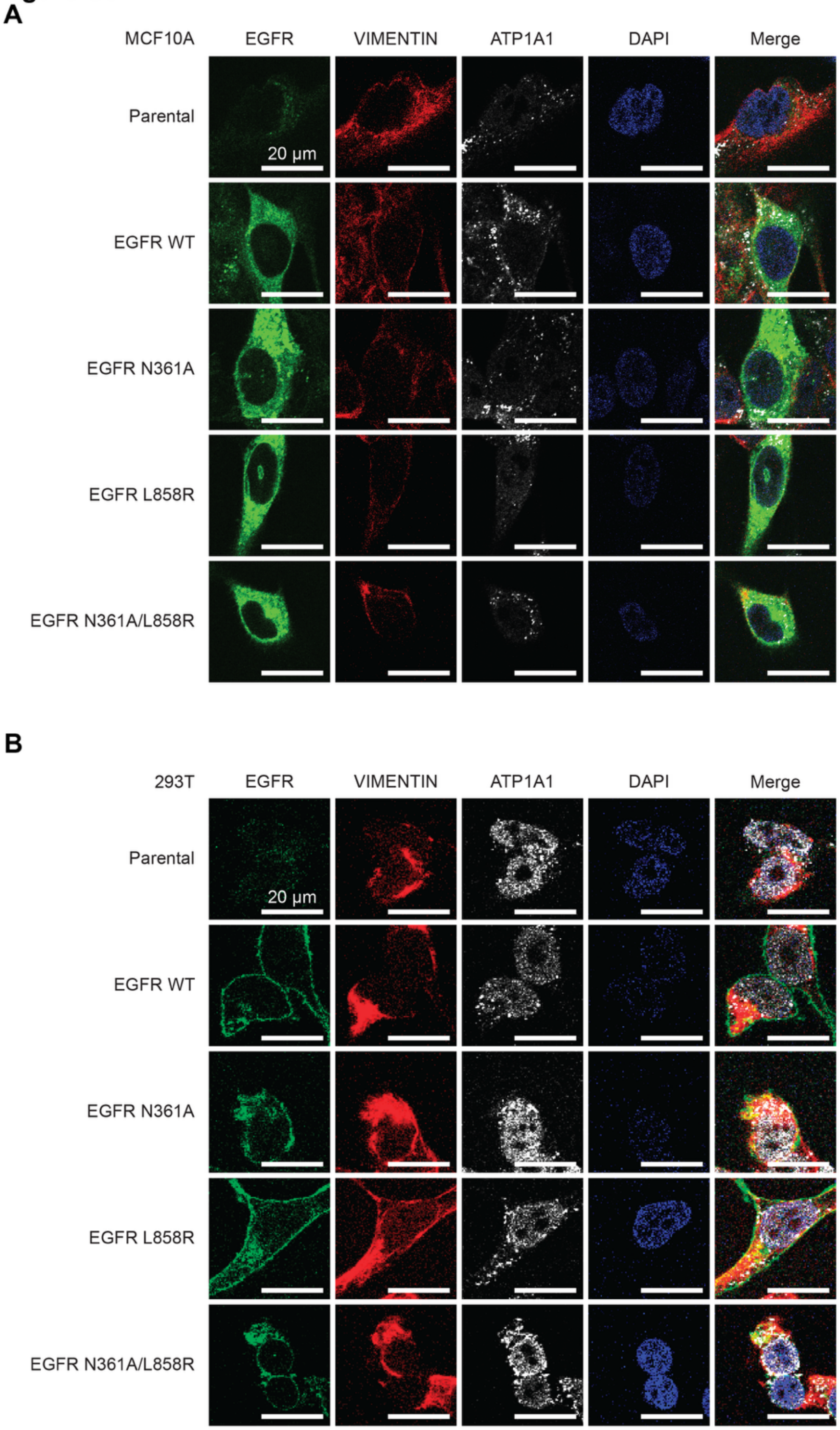

Figure S3. Immunofluorescence of EGFR mutants. A-B, Representative immunofluorescent microscopy images of MCF10A (A) or 293T (B) cells that were parental or overexpressed cDNAs of EGFR wild-type (WT), EGFR N361A, EGFR L858R, or a single construct containing two mutations EGFR N361A/L858R. Cells were stained for EGFR (green), vimentin (red), ATP1A1 (white), and DAPI (blue). Scale bars (red) represent 20 µm.

Figure S4

A

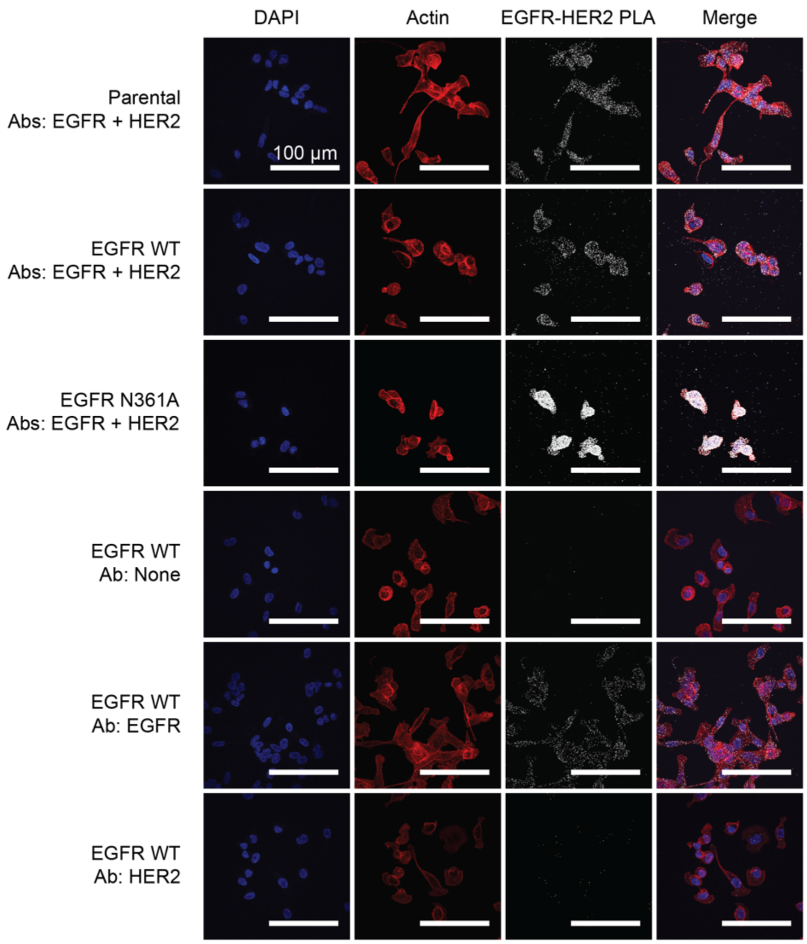

B

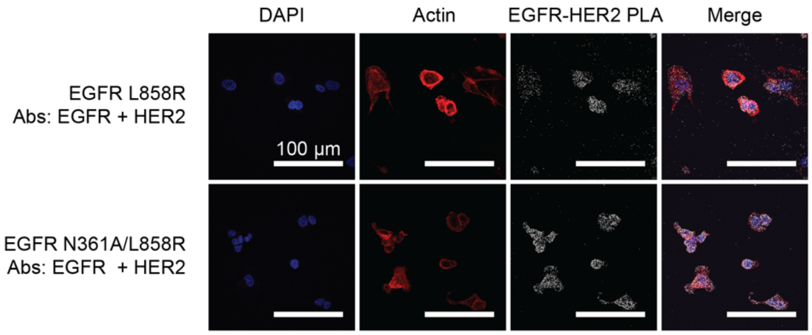

Figure S4. Proximity ligation assays from Figures 1B and 1D; Lower magnification fields are shown, scale bars represent 100 µM. In situ proximity ligation using antibodies against EGFR, HER2, or both are shown. DAPI and Actin stains were also performed on (A) MCF10A cells stably expressing EGFR WT, EGFR N361A, or parental cells; and (B) MCF10A cells stably expressing EGFR L858R or *cis* EGFR N361A/L858R.

Figure S5

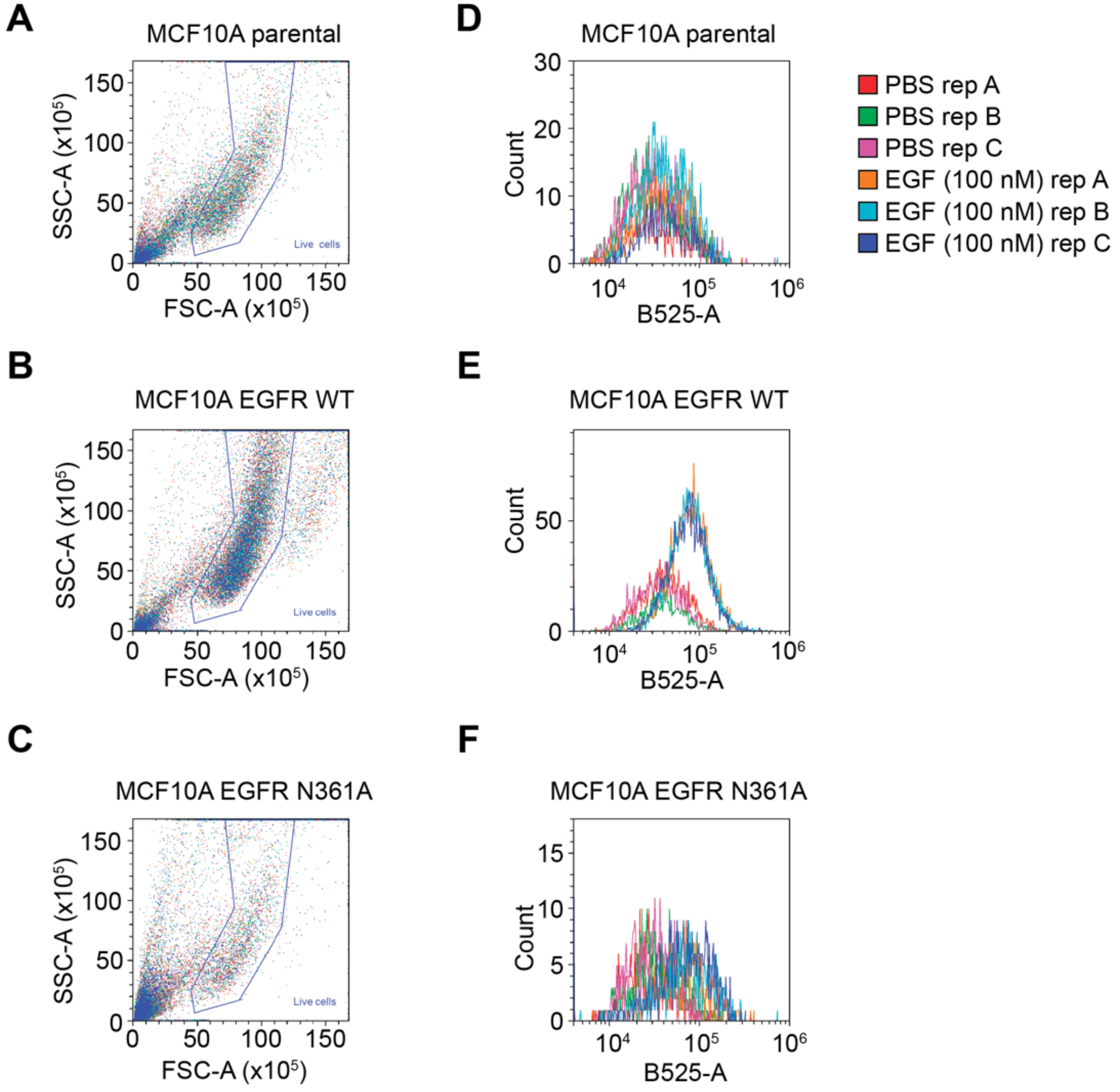

Figure S5. Flow cytometry data and gating corresponding to Figure 3C. MCF10A cells were treated with 100 nM fluorescein-EGF, followed by washes to remove unbound ligand; n=3 for each condition. **A-C,** Forward scatter vs. side scatter to gate live cells in MCF10A parental (A), EGFR WT (B), and EGFR N361A (C). **D-E,** Histograms of cell counts for cells treated with 100 nM fluorescein-EGF or control PBS. Histograms on the right use the corresponding live cell gates on the left. Quantifications of mean fluorescent intensity (MFI) are shown in Figure 3C.

Figure S6

A

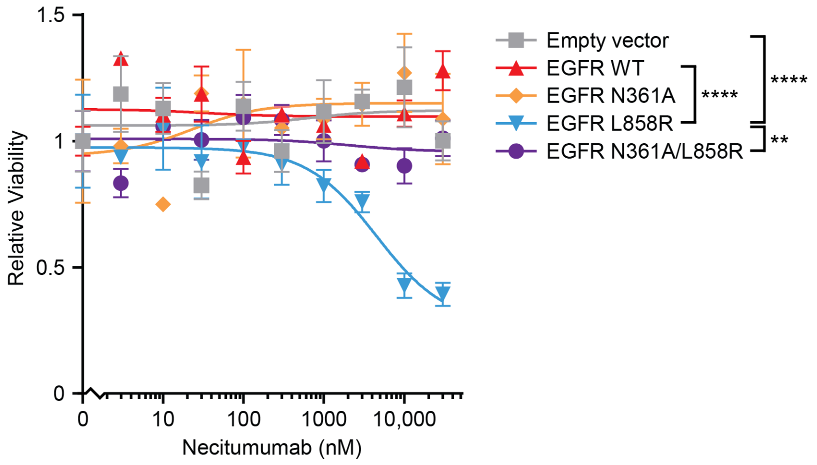

B

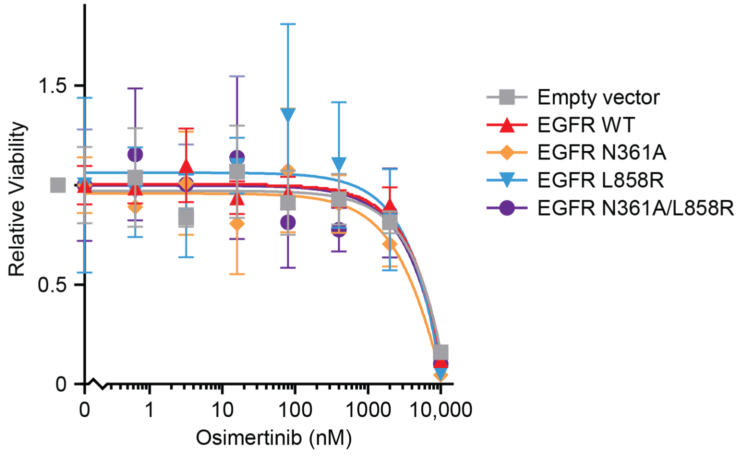

Figure S6. EGFR mutants modulate the effectiveness of EGFR inhibitors. Dose courses of relative viability of HEK-293T (293T) cells overexpressing the indicated cDNAs upon treatment with the indicated drug for 72 hours, measured by CellTiter-Glo. (**A),** 293T cells overexpressing Empty Vector, EGFR WT, EGFR N361A, EGFR L858R or EGFR N361A/L858R treated with indicated dose courses of necitumumab. (**B),** 293T cells overexpressing Empty Vector, EGFR WT, EGFR N361A, EGFR L858R, or EGFR N361A/L858R treated with the indicated doses of osimertinib. For all panels, n=3, ** $p < 0.01$, **** $p < 0.0001$.

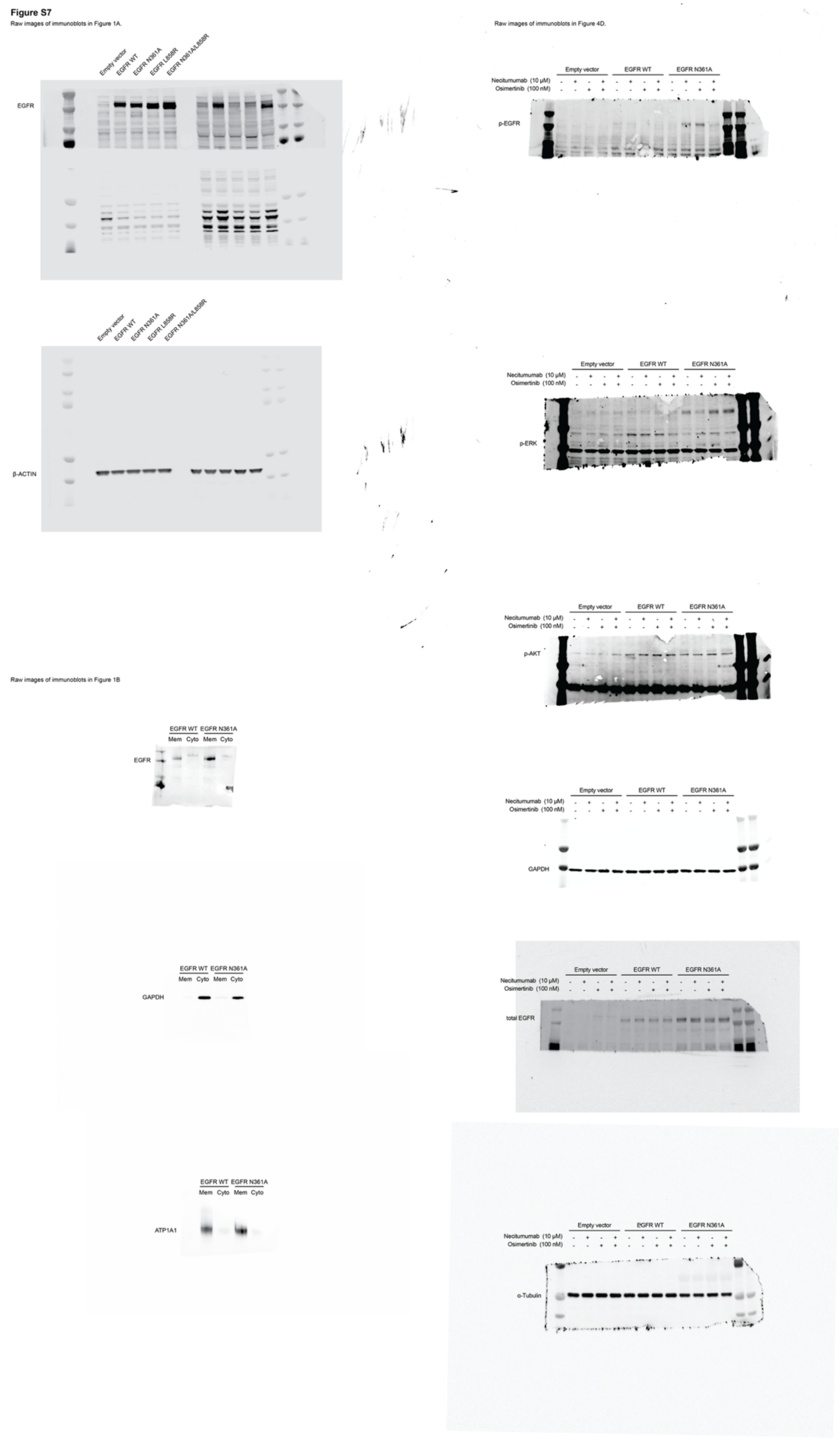

Figure S7. Uncropped immunoblot images for Figures 1A, 1B, and 4D.
